# Genotype frequency distributions of 28 SNP markers in two commercial lines and five Chinese native chicken populations

**DOI:** 10.1186/s12863-020-0815-z

**Published:** 2020-02-04

**Authors:** Jing-Jing Li, Long Zhang, Peng Ren, Ye Wang, Ling-Qian Yin, Jin-Shan Ran, Xian-Xian Zhang, Yi-Ping Liu

**Affiliations:** 1 Farm Animal Genetic Resources Exploration and Innovation Key Laboratory of Sichuan Province, Sichuan Agricultural University, Chengdu, 611130 Sichuan China; 2 Institute of Ecology, Key Laboratory of Southwest China Wildlife Resources Conservation (Ministry of Education), China West Normal University, Nanchong, 637009 Sichuan China

**Keywords:** Genetic marker, Growth trait, Egg laying trait, Native chicken

## Abstract

**Background:**

Modern breeding in the poultry industry mainly aims to produce high-performance poultry lines and breeds in two main directions of productivity, meat and eggs. To understand more about the productive potential of lowly selected Chinese native chicken populations, we selected 14 representative SNP markers strongly associated with growth traits or carcass traits and 14 SNP markers strongly associated with egg laying traits through previous reports. By using the MassArray technology, we detected the genotype frequency distributions of these 28 SNP markers in seven populations including four lowly selected as well as one moderately selected Sichuan native chicken populations, one commercial broiler line and one commercial layer line.

**Results:**

Based on the genotype frequency distributions of these 28 SNP markers in 5 native chicken populations and 2 commercial lines, the results suggested that these Chinese indigenous chicken populations have a relatively close relationship with the commercial broiler line but a marked distinction from the commercial layer line. Two native chicken breeds, Shimian Caoke Chicken and Daheng Broilers, share similar genetic structure with the broiler line.

**Conclusions:**

Our observations may help us to better select and breed superior domestic chickens and provide new clues for further study of breeding programs in local chicken populations.

## Background

The improvement of growth traits and egg laying traits is of major importance in modern poultry industry to enable producers to meet the increasing demands for meat and eggs [1]. Defining the molecular genetic basis of these economically important traits is a major task in chicken breeding research [2]. Heritability estimates showed that chicken growth traits and egg laying traits could be enhanced by genetic improvement [3, 4]. Most economically important traits are controlled by a series of genes or quantitative trait loci (QTLs) [2]. Following the rapid advancement of molecular genetic technologies and the availability of data, multiple researches have been performed to identify, map and analyze QTLs for application in marker-assisted selection (MAS) programs in chickens [5–8]. At present, there are two main strategies applied for detecting QTLs: association analysis using candidate genes and genome wide association study (GWAS) [9].

Chinese indigenous chickens possess a series of desirable meat qualities including greater tenderness and preferred flavors that are often favored by consumers [10, 11]. Besides, they are relatively disease-resistant and well-adapted to the harsh environments [12, 13]. However, unlike commercial chicken breeds that have undergone numerous generations of intense artificial selection, native chicken breeds have a relatively slow growth rate and low egg production [14]. Therefore, faster genetic improvement for higher growth or carcass traits and egg laying performances in Chinese native chicken breeds is expected to be achieved by breeding program [15]. Single nucleotide polymorphism (SNP) is a kind of efficient genetic marker based upon the variability at the nucleotide level [16]. Understanding the genotype frequency distributions of these SNPs that have significant associations with productive traits in Chinese native chicken populations will greatly uncover the productive potential for meat or egg propose of these birds.

To characterize the genetic variations and genetic relationships among different populations using DNA markers, a total of 28 identified SNP markers, including 14 growth or carcass traits associated loci and 14 egg laying related genome positions, were selected from the previous reports [17–33]. By using the MassArray technology, an Agena Bioscience MassARRAY System which is capable of efficiently genotyping tens to hundreds of SNPs with high accuracy, we detected the genotype frequency distributions of the 28 SNP markers in seven populations including four lowly selected as well as one moderately selected Sichuan native chicken populations, one commercial broiler line and one commercial layer line [34, 35]. The findings of the present study may lead to a better understanding of the relationship between native and commercial populations and will be helpful in the selection of superior native chickens.

## Results

### Genotype frequencies of the 28 SNP markers in the seven populations

**Fig. 1:**
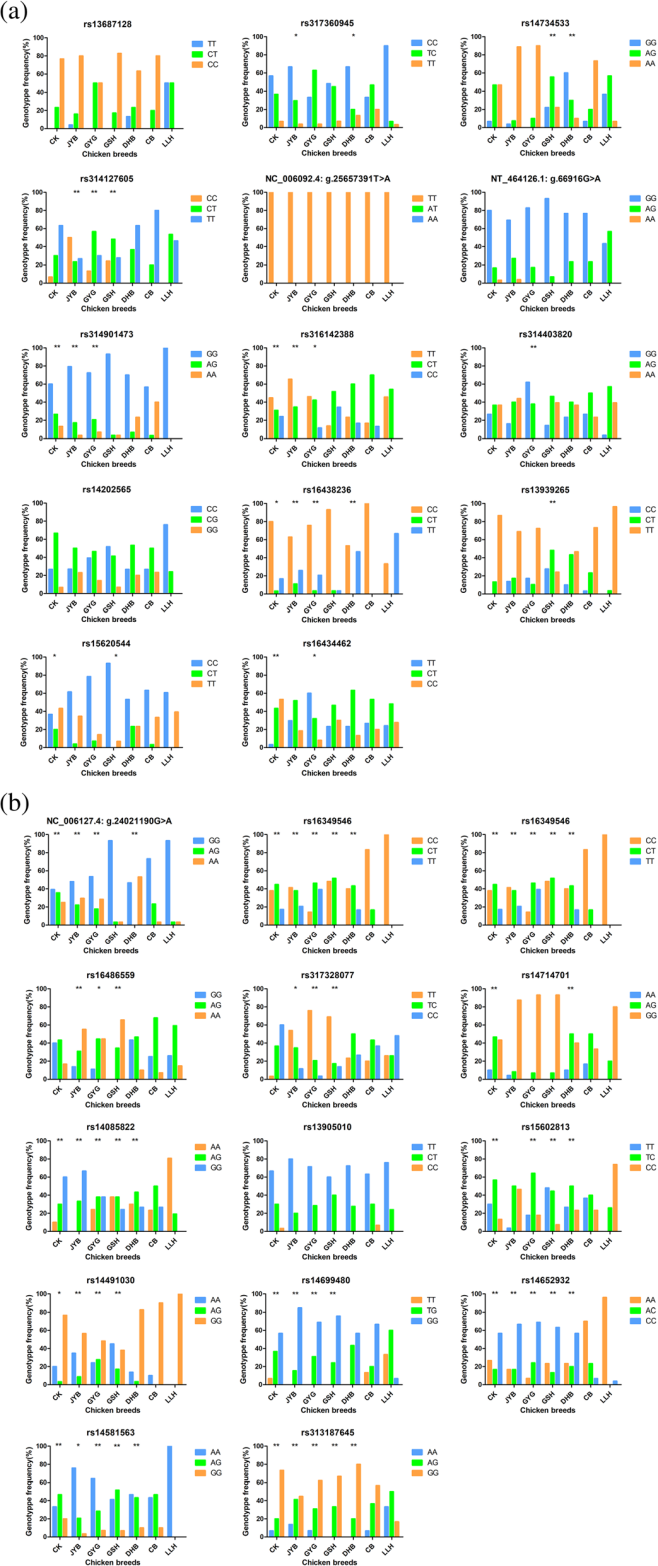
Comparisons of genotype frequency distributions on 28 SNP markers. **a** Genotype frequency distributions of 14 SNP markers associated with growth traits or carcass traits on seven populations. * on the top of each native chicken population represented significant difference between native chicken population and CB (*P*<0.05); ** represented extremely significant difference between native chicken population and CB (*P*<0.01). **b** Genotype frequency distributions of 14 SNP markers associated with egg production traits on seven populations. * on the top of each native chicken population represented significant difference between native chicken population and LLH (*P*<0.05); ** represented extremely significant difference between native chicken population and LLH (*P*<0.01)

Comparisons of genotype frequency distributions of the 28 SNPs markers in the seven chicken populations are shown in Fig. 1 and Mass spectrometry for 28 SNP markers are in Additional file 1: Figure S1 and Additional file 2: Figure S2. We did not detect the variation at NC_006092.4: g.25657391 T > A and no significant difference was found between the native chicken populations and the commercial broiler line at rs13687128 and rs14202565 (*P* > 0.05) (Fig. 1a). There were 5, 5, 6, 4, 3 SNP markers presenting significant difference (*P* < 0.05) between CK and CB, JYB and CB, GYG and CB, GSH and CB, DHB and CB, respectively (Fig. 1a). However, genotype frequencies of the broiler and layer populations appeared greatest difference in the 14 markers related to growth or carcass traits, with 4 SNP markers showing significant difference (*P* < 0.05) and 6 SNP markers showing extremely significant difference (*P* < 0.01) (Fig. 1a). Interestingly, the frequencies of the genotypes at most of the SNP markers associated with egg production traits exhibit extremely significant difference (*P* < 0.01) between LLH and the other six chicken populations. The frequencies of genotype GG, CC, AA, AA at rs14491030, rs16349546, rs14581563, rs315420959 in LLH reached 100%, with great genetic diversity in other Chinese indigenous chicken populations at these four SNP makers. These four specific genotypes may be advantageous for better egg performances in LLH due to intensive selection to achieve higher egg productivity.

### Clustering of the seven chicken populations

**Fig. 2:**
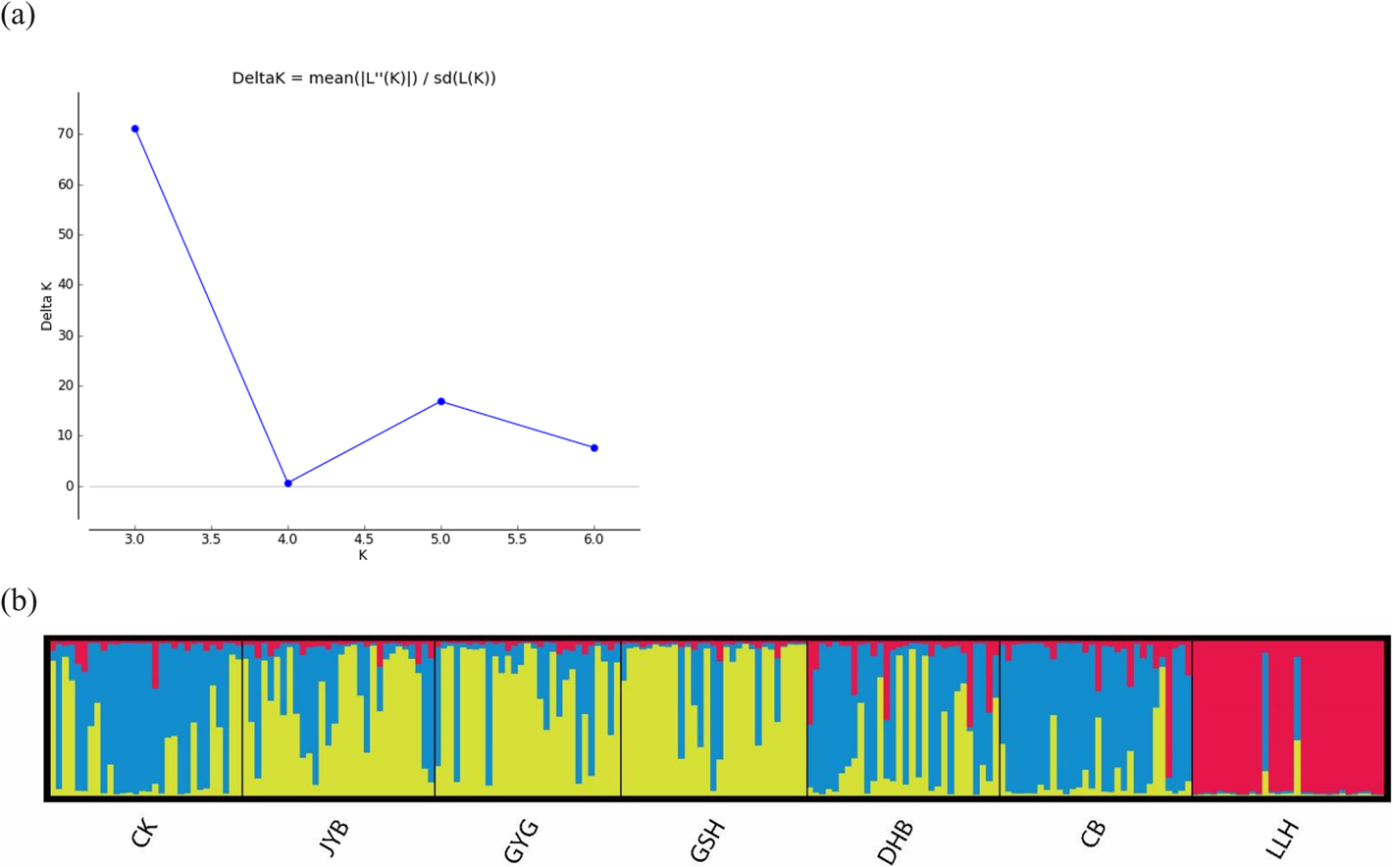
**a** Plots for detecting the number of K groups that best fit the data. The best value of K was 3 since Delta K was highest when K=3. **b** Population structure based on the genetic variation of 28 SNP markers inferred by Bayesian clustering. Each individual was shown as a thin vertical line partitioned into 3 colored components representing inferred membership in 3 genetic clusters

Consistent population structure with Bayesian cluster analysis among samples based on genotype frequencies of all 28 SNP markers was detected by STRUCTURE outputs. Results of the STRUCTURE analysis are given in Fig. 2 and plots for delta-K suggested that K = 3 was the optimum number of clusters among the full datasets (Fig. 2a) since the value of delta K was the highest when K was 3. Thus, we only displayed the population structure with 3 clusters here (Fig. 2). With this setting, commercial layer LLH formed a distinct cluster, which is consistent with the results of genotype frequencies. Besides, CK and DHB have similar population structure with CB. Other native chicken populations, including JYB, GYG, GSH, have share a similar population structure, which is distinctively different from both commercial broiler and layer.

## Discussion

Chicken growth and egg production traits are two economically important traits, which are determined by genetic, nutritional and environmental factors [18]. The genetic makeup ultimately has a fundamental influence and uncovering the molecular mechanism results in more efficient selections for meat or egg production in chicken populations [16]. To date, a large number of experiments have been performed successfully to identify QTLs for economically important traits in chickens [36]. After numerous generations of intense artificial selections, the frequencies of QTL alleles on these economy-related traits have increased in commercial lines [37]. However, no studies have been conducted to detect the genotype frequency of different native populations on these QTLs. In the present study, we investigated genetic diversities and relationships between and within seven chicken populations including two commercial breeds and five native breeds based on genotyping individuals at 28 SNP sites. Among the nearest genes or candidate genes correlated with growth traits, OCA2 and SLC27A1 genes take part in transmembrane transport process, while IGFBP2 and MC4R genes are involved in insulin secretion pathway. Besides, ATGL and SLC27A1 genes are capable of regulating lipid homeostasis including triglyceride catabolic or biosynthetic process. The other genes such as IGFBP2, OCA2, CAPN3, SETDB2 genes, play roles in cell growth, cell proliferation, cell activation and satellite cell activation, respectively (Additional file 3: Table S1). As for the potential candidate genes for egg production traits, BMP15, GREM1, GREM2 genes are important for the regulation of bone morphogenetic proteins (BMPs) signaling pathway. HMGCR and SEL1L gene take part in the pathways of lipid metabolic process. Besides, CBFB, NCAPG, LCORL and GTF2A1 genes are key regulators of transcription by RNA polymerase II. The consequence types of all SNP markers include missense variants, synonymous variants, intron variants, 3 prime UTR variants and intergenic variants (Additional file 4: Table S2).

Compared with the previous studies, our results further provided a verification of these genetic makers. For example, Nie et al., showed that rs13687128 is significantly associated with BW at 21, 35 days, SD at 63 days (*P* < 0.05) and highly significantly associated with BW at 28 days and ADG from 0 to 4 weeks of age (*P* < 0.01), and the C allele is advantageous for chicken growth traits (Table 1) [25]. While our findings suggested that there is no significant difference in the genotype frequency contribution between CB and native chicken breeds and the C allele is the dominating allele in commercial broiler line and native chicken breeds but completely absent in LLH (Fig. 1a). Similarly, Fig. 1a showed that the allele A and C were both absent in LLH at rs314901473 and rs316142388, respectively, while individuals with these two alleles were reported to have better growth performance at early growth stage in F2 resource population made up of the reciprocal cross between Gushi chicken and Anka broilers and at late growth stage in Jinghai yellow chickens, respectively (Table 1) [17, 22]. Although the frequency of allele A in CB was the highest among all seven chicken populations, the genotype GG was still the primary genotype in all chicken populations at rs314901473. Besides, Fig. 1a showed that the frequency of allele C at rs16438236 in CB reached 100% while the allele C was the minor allele in LLH population, which is consistent with previous finding that the allele C is the favorable allele for growth traits in a F2 resource population from the reciprocal crosses of Silky Fowl and White Plymouth Rock [31]. The frequency of the favorable allele G for carcass traits at 49 days at rs314403820 was higher than that of allele A in CB and GYG populations, whereas the allele A occurred more often than the allele G in the other chicken populations [18]. However, Nie et al., reported that individuals with the CT genotype at rs314127605 have the highest value for BW at 14, 21, 35, 42, 49, 56, 63, 70, 77 days in a F2 resource population made up of reciprocal cross between White Recessive Rock and Chinese Xinghua chickens (Table 1) [26]. In our study, the frequency of CT in the CB population was lower than that of CT in the other populations, and the TT genotype was the primary genotype (80%) in the commercial broiler line (Fig. 1a). The same condition appeared in the results of SNP markers associated with egg production traits in Fig. 1b. Han et al., showed that the egg number in chickens with the allele A is significantly higher than the individuals with the allele G at NC_006127.4: g24021190 G > A in F5 generation of Qing-Jiao-Ma breeding chickens (*P* < 0.01) (Table 2) [20] while the GG genotype (93.33%) occurred much more frequently than the other genotypes in our commercial layer line. Besides, Tyasi et al., showed that genotype GG and TC at rs16486559 and rs317328077 are both favorable for egg production at 56, 66 weeks in Chinese Dagu chicken breed (Table 2) while our results implied that the genotype AG and CC were found at a higher frequency in LLH at rs16486559 and rs317328077, respectively(Fig. 1b) [27]. These results collectively demonstrated that the identification of QTLs is probably population-specific and the conflicting observations may be caused by genetic background differentiation, geographic distances or the limited sample size of experimental populations [38].

**Table 1:** Summary of 14 SNP markers associated with growth traits or carcass traits

SNP marker	GGA^a^	Position^b^	Nearest gene^c^	consequence type^d^	SNP^d^	Traits^e^	Chicken populations^f^	Reference
rs13687128	1	92,866,047	POU1F1	synonymous variant	T/C	BW at 21, 35 days and SD at 63 days (*P* < 0.05), BW at 28 days and ADG from 0 to 4 weeks of age (*P* < 0.01)	F2 resource population made up of reciprocal cross between White Recessive Rock and Xinghua chickens	[25]
rs317360945	7	23,386,524	IGFBP2	synonymous variant	C/T	BW at 7, 14, 21, 28, 35, 42, 49, 56, 90 days, and CW, EW, BMW at 90 days (*P* < 0.01), LMW at 90 days (*P* < 0.05)	F2 resource population made up of reciprocal cross between White Recessive Rock and Xinghua chickens	[23]
rs14734533	4	70,143,452	TBC1D1	3 prime UTR variant	G/A	BW, CW, EW, SEW and LMW at 90 days (*P* < 0.05)	Erlang Mountainous chickens	[28]
rs314127605	5	15,760,767	ATGL	missense variant	C/T	BW at 14, 21, 35, 63, 70, 77 days, CFW and AFW (*P* < 0.05), BW at 42, 49, and 56 days (*P* < 0.01)	F2 resource population made up of reciprocal cross between White Recessive Rock and Chinese Xinghua chickens	[26]
NC_006092.4: g.25657391 T > A	5	25,657,391	CAPN3	intron variant	T/A	BW, EP, BMP at 90 days (*P* < 0.05)	five commercial pure lines (S01, S02, S03, S05, and D99) and 4 Chinese native breeds (Huiyang Huxu chicken, Qingyuan Ma chicken, Caoke chicken and Mountainous black-bone chicken)	[33]
NT_464126.1: g. 66916G > A	30	66,916	SLC27A1	missense variant	G/A	LW, CW, SEW, EW and BMWP at 91 days (*P* < 0.05)	eight meat-type quality chicken populations including S01, S02, S03, S05, S06, D99, S05 × S01, and S06 × S01 of Dahen chicken	[29]
rs314901473	1	14,235,966	PBEF1	synonymous variant	G/A	BW at 4, 6 weeks, and SL, BWLR and BFD at 4 weeks (*P* < 0.05), and BBL at 4 weeks (*P* < 0.01)	F2 resource population made up of reciprocal cross between Gushi chicken and Anka broilers	[22]
rs316142388	1	169,208,798	SETDB2	intron variant	T/C	BW at 12, 14, 16 weeks (*P* < 0.05)	Jinghai yellow chickens	[17]
rs314403820	3	67,466,233	FOXO3	intron variant	G/A	CW, SEW, EW and BMW at 49 days (*P* < 0.05)	White Recessive Rock and Xinghua chickens	[18]
rs14202565	2	69,322,385	MC4R	missense variant	G/C	BW, CW, SHL at 7 weeks (*P* < 0.05)	F6 generation of Arbor Acres grandsire line	[24]
rs16438236	4	77,857,657	87 kb U BOD1L^g^	intergenic variant	C/T	BW at 10, 11, 12 weeks and ADG from 6 weeks to 12 weeks of age (*P* < 0.01)	F2 resource population made up of reciprocal crosses of Silky Fowl and White Plymouth Rock	[31]
rs13939265	1	130,801,788	OCA2	intron variant	C/T	BW at 11 weeks and 12 weeks (*P* < 0.01)	F2 resource population made up of reciprocal crosses of Silky Fowl and White Plymouth Rock	[31]
rs15620544	4	77,233,676	FBXL5	intron variant	C/T	BW at 10 weeks (*P* < 0.01)	F2 resource population made up of reciprocal crosses of Silky Fowl and White Plymouth Rock	[31]
rs16434462	4	73,027,691	—^h^	intergenic variant	T/C	BW at 11, 12 weeks and ADG from 6 weeks to 12 weeks of age (*P* < 0.01)	F2 resource population made up of reciprocal crosses of Silky Fowl and White Plymouth Rock	[31]

^a^‘GGA’ refers to chromosome of *Gallus gallus*

^b^Position was based on chicken genome version of Gallus_gallus-5.0

^c^The nearest genes were obtained from the NCBI database or the reference in the rightmost column

^d^The consequence types and SNP were obtained from Ensemble database or the reference in the rightmost column

^e^The traits were obtained from the reference in the rightmost column and showed strong association with SNP marker in the leftmost column

^f^The chicken populations refer to the experimental material of the reference

^g^‘U’ indicates that the SNP is upstream of the gene

^h^‘—’ indicates that there was no nearest gene of rs16434462

**Table 2:** Summary of 14 SNP markers associated with reproduction traits

SNP marker	GGA	Position	Nearest gene	consequence type	SNP	Traits	Chicken populations	Reference
NC_006127.4: g24021190 G > A	Z	24,021,190	HMGCR	intron variant	G/A	EN at 300 days (*P* < 0.01)	F5 generation of Qing-Jiao-Ma breeding chickens	[20]
rs16349546	4	1,874,976	BMP15	synonymous variant	C/T	AFE, EWFE, EW at 43 weeks, EP at 43 weeks, EP at 46 weeks and EP at 48 weeks (*P* < 0.05)	LaiWu Black chickens	[21]
NC_006096.4: g22545152 G > C	9	22,545,152	RARRES1	nonsynonymous variant	G/C	EPR from 169 day to 280 day of age(*P* < 0.01)	F2 resource population crossed by White Leghorn males and Rhode Island Red females	[9]
rs16486559	5	30,579,826	GREM1	synonymous variant	G/A	EP at 43, 57, 66 weeks (*P* < 0.05)	Chinese Dagu chickens	[27]
rs317328077	3	36,305,243	GREM2	synonymous variant	T/C	EP at 30, 57, 66 weeks (*P* < 0.05)	Chinese Dagu chickens	[27]
rs14714701	7	20,960,237	KCNH7	intergenic variant	A/G	FEW (*P* < 0.01)	Jinghai Yellow Chicken	[19]
rs14085822	3	12,329,322	CDC42BPA	intron variant	A/G	EW at 300 days(*P* < 0.01)	Jinghai Yellow Chicken	[19]
rs13905010	1	92,440,932	GJA5	intron variant	T/C	EN between the age of 300 and 462 days (*P* < 0.01)	Jinghai Yellow Chicken	[19]
rs15602813	11	2,417,179	CBFB	intron variant	T/C	EN between the age of 300 and 462 days (*P* < 0.01)	Jinghai Yellow Chicken	[19]
rs14491030	4	76,458,342	NCAPG	missense variant	A/G	EN between the age of 26 and 28 weeks (*P* < 0.01), EN between the age of 42 and 46 weeks (*P* < 0.01)	six generations of a purebred brown egg layer line	[30]
rs14699480	4	76,411,761	LCORL	intron variant	T/G	EN between the age of 26 and 28 weeks (*P* < 0.01)	six generations of a purebred brown egg layer line	[30]
rs14652932	8	23,991,868	FAF1	intron variant	A/C	EN between the age of 26 and 28 weeks (*P* < 0.01)	six generations of a purebred brown egg layer line	[30]
rs315420959	5	41,036,029	21 kb U SEL1L	intergenic variant	A/G	EN between the age of 21 and 40 weeks (*P* < 0.01)	F2 resource population made up of reciprocal crosses between White Leghorn and Dongxiang Blue-shelled chicken	[32]
rs313187645	5	40,875,680	GTF2A1	intron variant	G/A	EN between 21 and 40 weeks of age(*P* < 0.01)	F2 resource population made up of reciprocal crosses between White Leghorn and Dongxiang Blue-shelled chicken	[32]

As is shown in Fig. 2, the STRUCTURE analysis exhibited a distinct clustering of LLH population, which suggested that more work is still needed for the genetic improvement of Sichuan native chicken breeds for egg purpose based on the SNP markers we selected here. Furthermore, CK, DHB and CB populations appeared to be grouped separately, suggesting that these three chicken breeds share closer genetic relationship based on these genotype frequencies of the 28 SNP markers. In fact, CK and DHB present more appropriate for meat propose than the other three native chicken breeds according to their productive performance (Table 3). DHB is a moderately selected chicken breed for meat production in China and the selective breeding for this breed seems to have already achieved a great genetic improvement based on our results. Other native chicken populations, including JYB, GYG, GSH, share a similar population structure in STRUCTURE analysis, which is distinctively different from both of the commercial broiler and layer line. This is consistent with the fact that these native chicken populations have been under low selection and breeding. A great diversity was also observed between commercial chicken populations and native populations in the previous reports. Using 29 autosomal microsatellite markers, Mtileni et al. demonstrated that all the domestic chickens were diverse from the commercial lines and the village chicken formed a single cluster while commercial populations formed separate and distinct clusters [39]. Besides, in a study that employed 30 microsatellite markers, 15 chicken population samples collected from Kenya, Uganda, Ethiopia and Sudan were used to detect genetic diversity and the results showed a closely genetic relationship among these indigenous chickens but a marked distinction from commercial breeds [40].

**Table 3:** Morphological description of Chinese native chicken populations

Chicken breeds	Source	BW at 90 days of age(kg)^a^	BW at 120 days of age(kg)	BW at 150 days of age(kg)	EN at 300 days of age
JYB	Wanyuan Hengkang Agricultural Development Company	1.29 ± 0.15	1.37 ± 0.15	1.50 ± 0.06	95
GYG	Sichuan Tianguan Ecological Agriculture and Animal Husbandry Company	0.93 ± 0.06	1.41 ± 0.09	1.58 ± 0.14	90
CK	Shimian Pengcheng Breeding Company	2.30 ± 0.24	2.89 ± 0.06	3.89 ± 0.15	120
GSH	Sichuan Agricultural University Breeding Farm	0.89 ± 0.01	1.28 ± 0.02	1.48 ± 0.05	115
DHB	Sichuan Daheng Poultry Breeding Company	1.85 ± 0.03	2.52 ± 0.02	3.11 ± 0.07	90

^a^The values in the tables are mean ± SE, which are recorded from local poultry breeding companies and we only showed the body weight of male chickens

Chinese chicken breeds exhibit a wide spectrum of phenotypic and morphologic properties, harboring valuable genetic resource of functional mutations affecting a wide range of properties [41]. On the basis of maintaining the uniqueness of these breeds, the rich genetic diversity requires effective characterization for breeding and conservation purposes [42]. The success of these strategies is closely hinged on a good knowledge of the phenotypic and genetic architecture of indigenous chicken populations. Applying these molecular genetic markers into lowly selected Chinese native chicken breeds with MAS will greatly enhance the intensity of selection and efficiently accelerate great genetic improvement for growth and egg production traits. At the same time, it will bring great convenience to animal breeding work, such as guiding the rapid screening of breeding materials, designing molecular combination schemes for target traits, and evaluating the molecular progress of breeding programs.

In summary, our observations provided new clues to understand the productive potential of Chinese native chicken and may benefit the further study of economically important traits and breeding programs in Chinese local chicken populations.

## Conclusions

Based on the genotype frequency distributions of 28 SNP markers, the great diversity is observed between commercial chicken populations and native populations. Besides, five Chinese indigenous chicken populations have a relatively close relationship with the commercial broiler line but a marked distinction from the commercial layer line. Two native chicken breeds, CK and DHB, share similar genetic structure with the commercial broiler line.

## Methods

### Selection and summary of 28 SNP markers

Based on multiple previous reports [17–33], we have chosen 14 characteristic SNP markers strongly associated with growth traits or carcass traits (*P* < 0.05) including rs13687128, rs317360945, rs14734533, rs314127605, NC_006092.4: g.25657391 T > A, NT_464126.1: g.66916G > A, rs314901473, rs316142388, rs314403820, rs14202565, rs16438236, rs13939265, rs15620544, rs16434462 and 14 characteristic SNP markers with strongly association with egg production traits (*P* < 0.05) including NC_006127.4: g.24021190G > A, rs16349546, NC_006096.4: g22545152 G > C, rs16486559, rs317328077, rs14714701, rs14085822, rs13905010, rs15602813, rs14491030, rs14699480, rs14652932, rs315420959 and rs313187645 for genotype frequency analysis. The specific information of the SNP markers is given together with their genetic background and corresponding traits in Tables 1 and 2. These SNP markers are all identified by candidate gene method and GWAS from previous reports and the information including positions, nearest genes, consequence types of these SNP markers were obtained from the National Center for Biotechnology Information (NCBI) database (https://www.ncbi.nlm.nih.gov/) or Ensemble database (http://www.ensembl.org). The growth traits mainly included body weight (BW) and average daily weight gains (ADG) at different stages, while the carcass traits mainly included carcass weight (CW), eviscerated weight (EW), eviscerated percentage (EP), semi-eviscerated weight (SEW), slanting length (SL), shank diameters (SD), shank length (SHL), chest width (CHW), cingulated fat width (CFW), abdominal fat pad weight (AFW), breast muscle weight (BMW), leg muscle weight (LMW), breast muscle percentage (BMP), breast muscle water loss rate (BWLR), breast bone length (BBL) and breast muscle fiber density (BFD). The laying traits mainly included egg number (EN), egg production rate (EPR), first egg weight (FEW), age at first egg (AFE), egg production (EP) and egg weight (EW) at different age. The gene ontology (Go) annotations of the nearest genes associated with growth traits (Additional file 3: Table S1.) were summarized from the GeneCards database (https://www.genecards.org/) and the Uniprot database (https://www.uniprot.org/), while these related with egg laying traits are in Additional file 4: Table S2.

### Sampling and genomic DNA extraction

A total of 210 samples from seven chicken breeds were used to screen the allelic variation of the selected loci. The chicken breeds are Caoke Chicken (CK), Guanyuan Grey Chicken (GYG), Jiuyuan Black Chicken (JYB), Green Shell Hen (GSH), Daheng Broiler (DHB), Cobb Broiler (CB) and Lohman Laying Hen (LLH) (Table 4). Each population was composed of 30 unrelated individuals, which were gifted with permissions from local poultry breeding companies. The morphological description of CK, GYG, JYB, GSH and DHB were collected from local poultry breeding companies (Table 3). The samples of each breed line were selected from one site only. Venous blood samples were collected from 210 samples under the wing of the chickens for genomic DNA extraction. Total genomic DNA was isolated with the TIANamp Blood DNA Kit in accordance with the manufacturer’s instructions, and then stored at − 20 °C, prepared for PCR amplification. Birds were released after blood extraction and the entire study was approved by the Committee on the Care and Use of Laboratory Animals of the State-level Animal Experimental Teaching Demonstration Center of Sichuan Agricultural University [43].

**Table 4:** The characterization of 7 chicken populations in this study

Populations	Abbreviation	Population size	Type
Jiuyuan Black Chicken	JYB	30	Sichuan native breed (lowly selected)
Guanyuan Grey Chicken	GYG	30	Sichuan native breed (lowly selected)
Shimian Caoke Chicken	CK	30	Sichuan native breed (lowly selected)
Green Shell Hens	GSH	30	Sichuan native breed (lowly selected)
Daheng Broilers	DHB	30	Sichuan native breed (moderately selected for meat purpose)
Cobb Boilers	CB	30	Commercial boiler line (highly selected for meat purpose)
Lohman Laying Hens	LLH	30	Commercial layer line (highly selected for egg purpose)

### Polymerase chain reaction (PCR) and genotyping

PCR primers of all 28 SNP markers were designed with Assay Desigh 3.1 software and synthesized by Beijing Huada gene laboratory. PCR was carried out in a final volume of 5 μL containing 1 μL (20 ng/μL) of DNA template and 4 μL PCR master mix (Additional file 5: Table S3). The PCR amplifcation conditions were as, initial denaturation at 94 °C for 5 min, followed by 45 cycles of 94 °C for 20s, 56 °C for 30s, and primer extension at 72 °C for 180 s. The PCR products were disposed with shrimp alkaline phosphatase (SAP) to remove remaining deoxyribonucleotide triphosphate (dNTP) (Additional file 6: Table S4). A total volume of 7 μL containing 5 μL products and 2 μL SAP mix were put in PCR device with the amplifcation condition of 37 °C for 20 min, 85 °C for 5 min. After the sufficient dispose of SAP, EXTEND mix was compounded for single base extension (Additional file 7: Table S5). A total volume of 9 μL containing 2 μL EXTEND mix and 7 μL SAP+PCR reaction products was into extension condition of 94 °C for 30s, 94 °C for 5 s, followed by 45 cycles of 94 °C for 5 s, 52 °C for 5 s, 80 °C for 5 s, and a final extension at 72 °C for 180 s. Finally, the PCR products were spotted into SpectroCHIP bioarray with the automatic instrument. MALDI-TOF-MS (SpectroREADER, Sequenom) was used to detect the chips. The plate data and scatter plot of data were processed by TYPER4.0 software.

### Statistical analysis

The genotype frequencies of the 28 SNP markers in seven chicken breeds were calculated by direct counting method [40]. The histograms of genotypic frequencies were made by Graphpad Prism 7, as we previously described [44, 45]. Comparisons of genotype frequencies between the Sichuan native chicken population and commercial broiler line or layer line were analyzed by Fisher’s exact test in R software v3.5.3. Clustering of individuals based on the genotype of 28 SNP markers was performed by STRUCTURE v2.3.4, which can assess the likelihood values of partitioning their data into different numbers of clusters (K). A Monte Carlo Markov chain was run for K = 2 to K = 8 with a run length of burin period of 200,000 and a number of MCMC reps after burin of 1200,000. For each K-value, 10 repeated runs were performed to calculate the mean L (K) [46]. The results generated by the program STRUCTURE were compressed and uploaded to a web-based program named STRUCTURE HARVESTER (http://taylor0.biology.ucla.edu/structureHarvester/), which is used to detect the best number of clusters by Evanno method and generated indfiles and popfiles for use with CLUMPP [47, 48]. CLUMPP aligned cluster assignment across replicate analyses and the results were visualized using DISTRUCT [49].

## Supplementary information

**Additional file 1: Figure S1.** Mass spectrometry for 14 SNP markers associated with growth traits or carcass traits.

**Additional file 2: Figure S2.** Mass spectrometry for 14 SNP markers associated with egg production traits.

**Additional file 3: Table S1.** Go annotation of nearest genes associated with growth traits or carcass traits.

**Additional file 4: Table S2.** Go annotation of nearest genes associated with reproduction traits.

**Additional file 5: Table S3.** The composition of PCR master mix.

**Additional file 6 Table S4.** The composition of SAP mix.

**Additional file 7: Table S5.** The composition of Extend mix.

## Data Availability

All data generated or analyzed during this study are included in this published article.

## Abbreviations

ADG: Average daily weight gains
AFE: Age at first egg
AFW: Abdominal fat pad weight
ATGL: Adipose triglyceride lipase
BBL: Breast bone length
BFD: Breast muscle fiber density
BMP: Breast muscle percentage
BMP15: Bone morphogenetic protein 15
BMW: Breast muscle weight
BOD1L: Biorientation of chromosomes in cell division 1 like 1
BW: Body weight
BWLR: Breast muscle water loss rate
CAPN3: Calpain-3
CB: Cobb Broiler
CBFB: Core-binding factor beta subunit
CDC42BPA: CDC42 binding protein kinase alpha
CFW: Cingulated fat width
CHW: Chest width
CK: Caoke Chicken
CW: Carcass weight
DHB: Daheng Broiler
dNTP: deoxyribonucleotide triphosphate
EN: Egg number
EP: Egg production
EP: Eviscerated percentage
EPR: Egg production rate
EW: Egg weight
EW: Eviscerated weight
FAF1: Fas associated factor 1
FBXL5: F-Box And Leucine Rich Repeat Protein 5
FEW: First egg weight
FOXO3: Forkhead box O3
GJA5: Gap junction alpha-5
Go: Gene ontology
GREM1: Gremlin-1
GREM2: Gremlin-2
GSH: Green Shell Hen
GTF2A1: General transcription facter IIA subunit 1 variant 1
GWAS: Genome wide association study
GYG: Guanyuan Grey Chicken
HMGCR: 3-hydroxy-3-methylglutaryl coenzyme A reductase
IGFBP2: Insulin-like growth factor-binding protein 2
JYB: Jiuyuan Black Chicken
KCNH7: Potassium voltage-gated channel subfamily H member 7
LCORL: Ligand dependent nuclear receptor corepressor-like protein
LLH: Lohman Laying Hen
LMW: Leg muscle weight
MAS: Marker-assisted selection
MC4R: Melanocortin 4 receptor
NCAPG: Non-SMC condensin I complex subunit G
OCA2: Oculocutaneous albinism II
OCX-32: Ovocalyxin-32
PBEF1: Nicotinamide phosphoribosyltransferase
PCR: Polymerase chain reaction
POU1F1: POU class 1 homeobox 1
QTL: Quantitative trait loci
SAP: Shrimp alkaline phosphatase
SD: Shank diameters
SEL1L: ERAD E3 ligase adaptor subunit
SETDB2: SET domain bifurcated 2
SEW: Semi-eviscerated weight
SHL: Shank length
SL: Slanting length
SLC27A1: Solute carrier family 27 member 1
SNP: Single nucleotide polymorphism
TBC1D1: TBC1 domain family member 1
