# Supplementary material for: Genotype frequency distributions of 28 SNP markers in two commercial lines and five Chinese native chicken populations
Source: BMC Genet. 2020 Feb 4;21:12. doi: 10.1186/s12863-020-0815-z (PMC7001339; doi:10.1186/s12863-020-0815-z)

Additional file 1: Figure S1. Mass spectrometry for 14 SNP markers associated with growth traits or carcass traits. The number in brackets indicated the number of individuals with respective genotype.


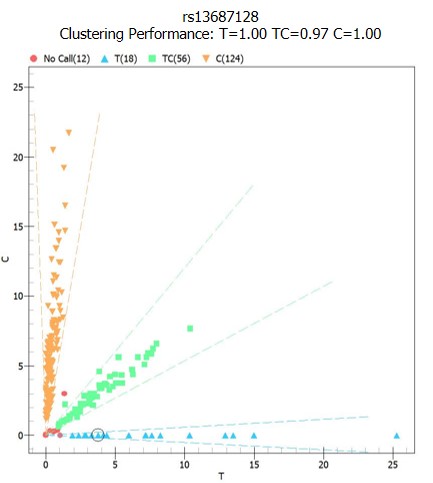

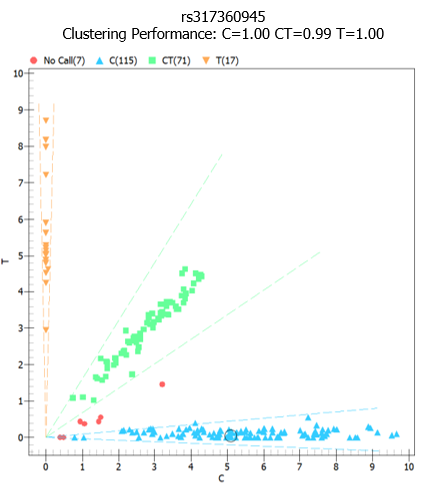

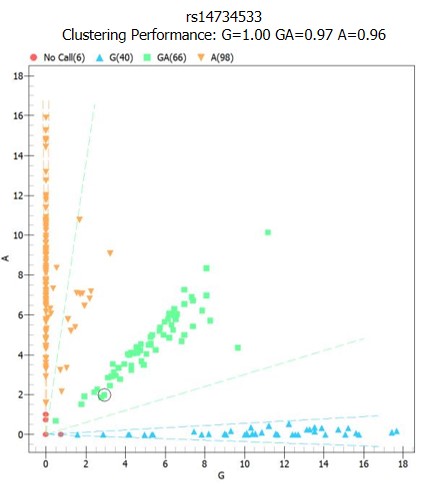

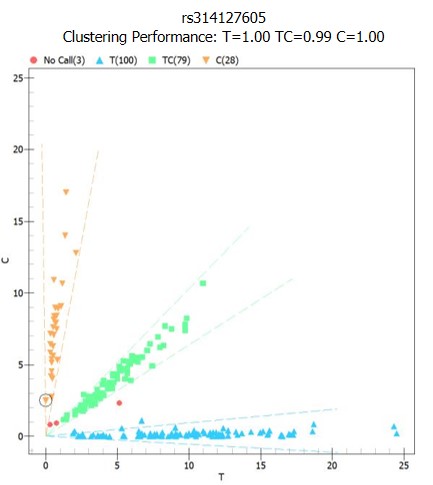

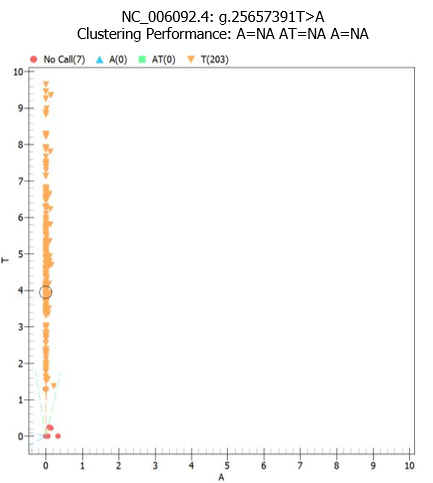

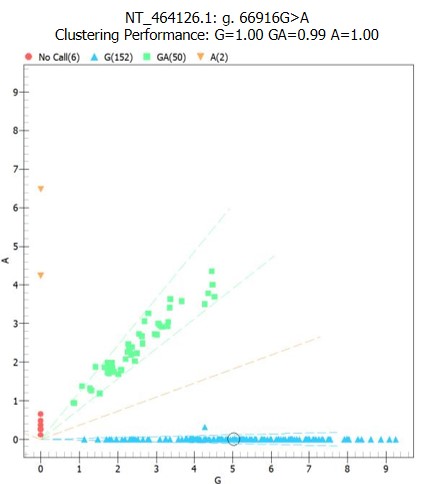

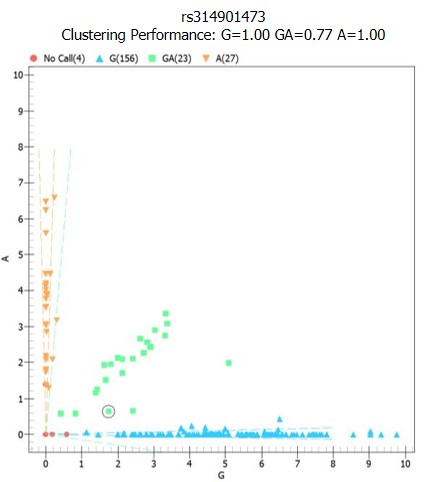

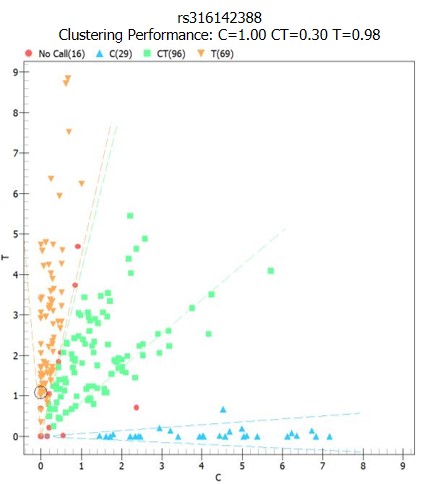

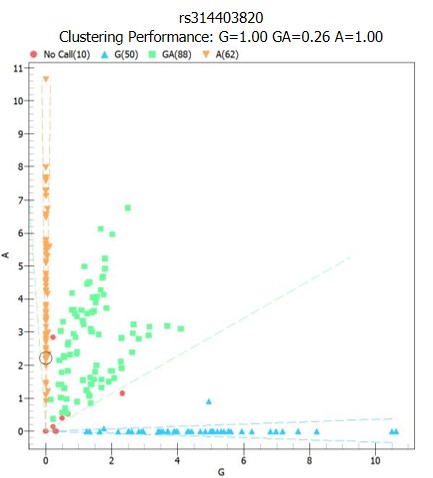

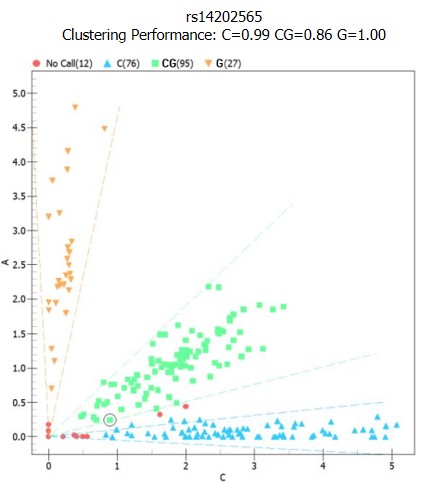

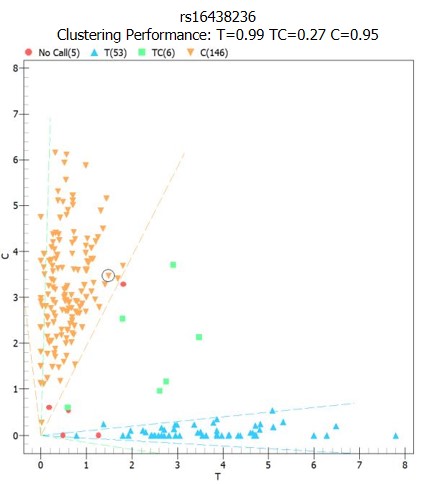

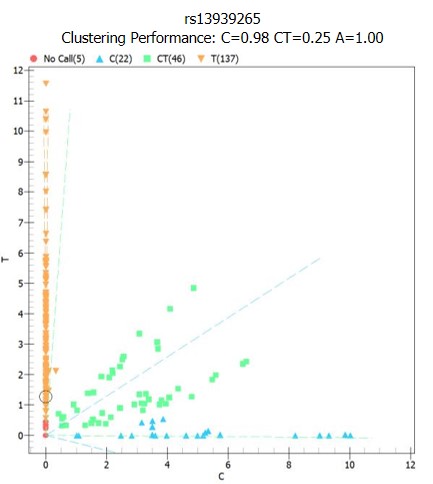

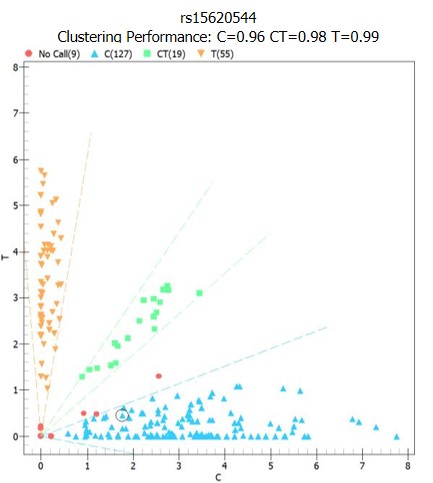

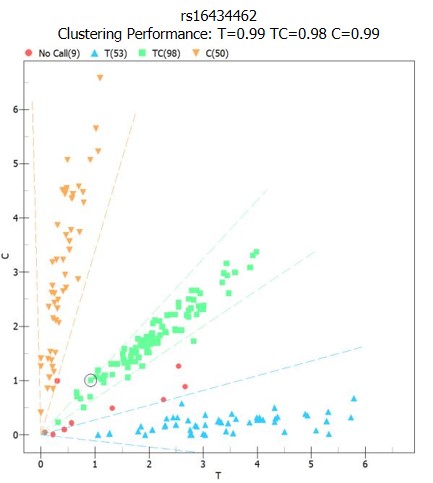

Supplement: Supplementary file 1 — Additional file 1: Figure S1. Mass spectrometry for 14 SNP markers associated with growth traits or carcass traits. [file 12863_2020_815_MOESM1_ESM.docx]
