# Supplementary material for: Genotype frequency distributions of 28 SNP markers in two commercial lines and five Chinese native chicken populations
Source: BMC Genet. 2020 Feb 4;21:12. doi: 10.1186/s12863-020-0815-z (PMC7001339; doi:10.1186/s12863-020-0815-z)

Additional file 2: Figure S2. Mass spectrometry for 14 SNP markers associated with egg production traits.


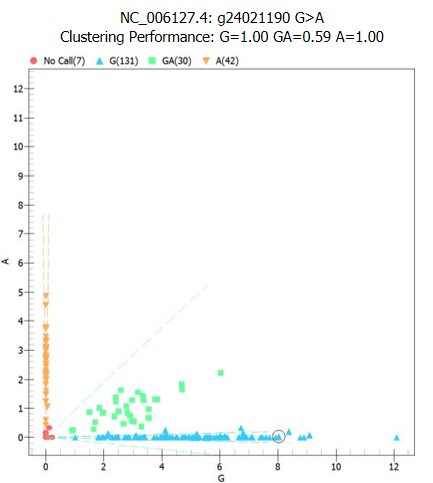

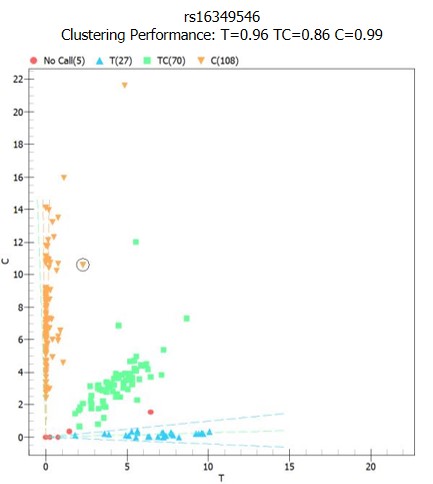

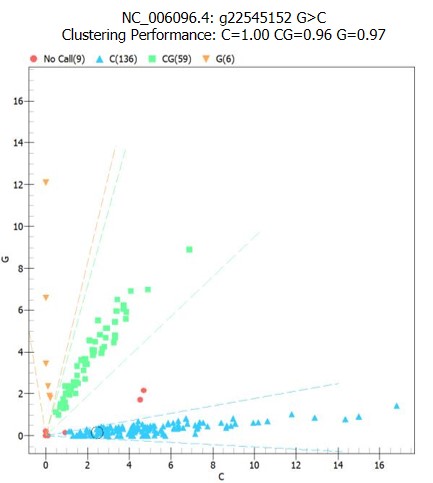

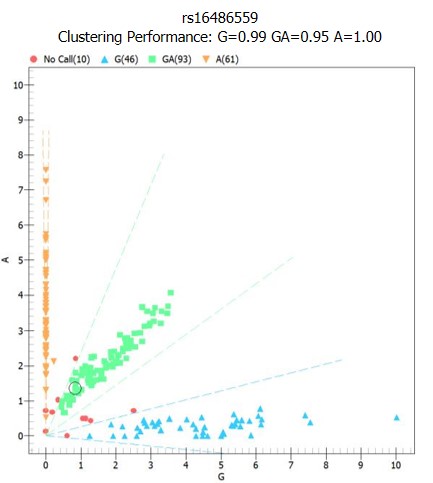

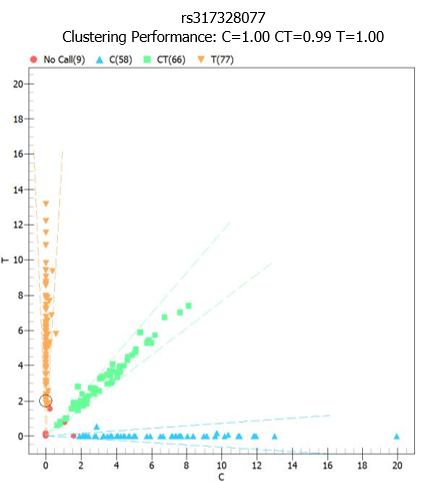

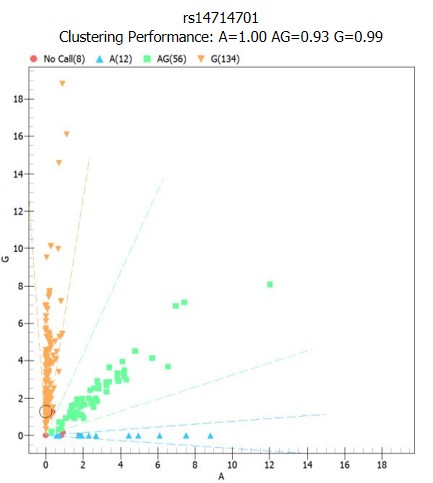

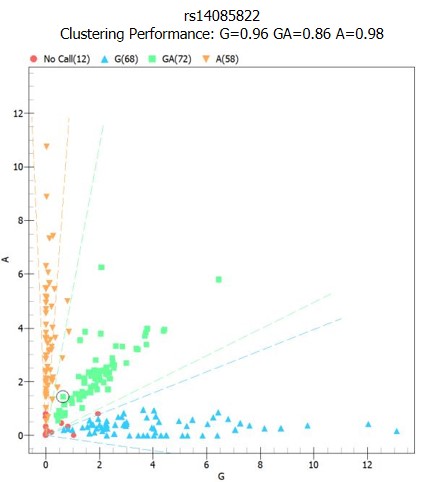

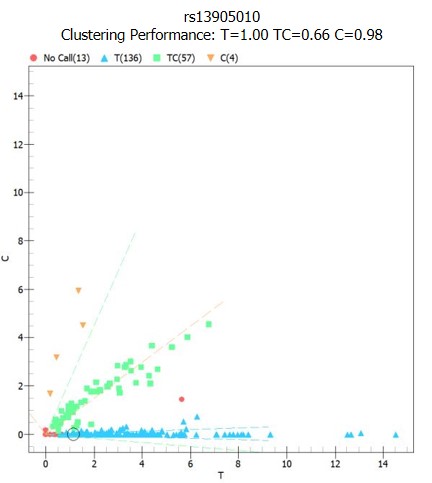

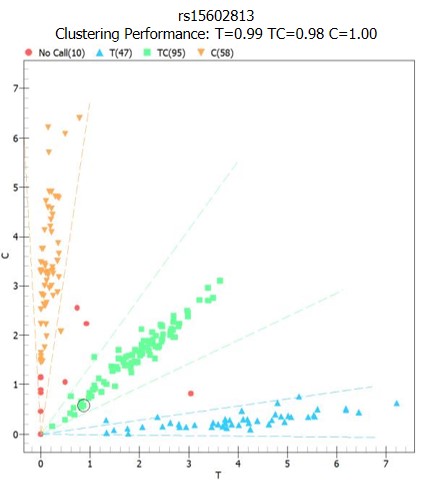

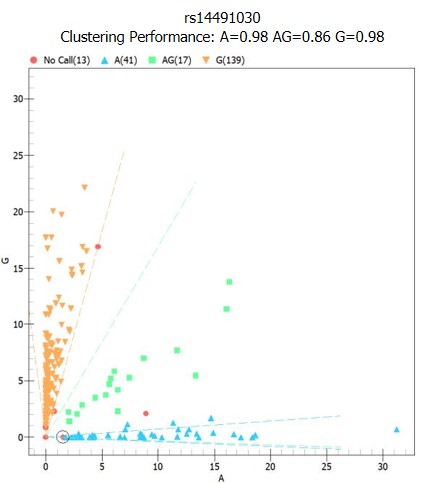

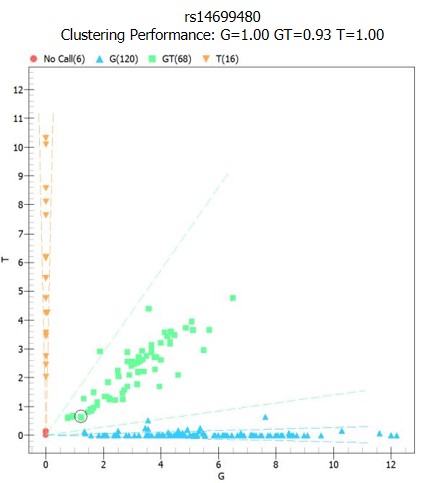

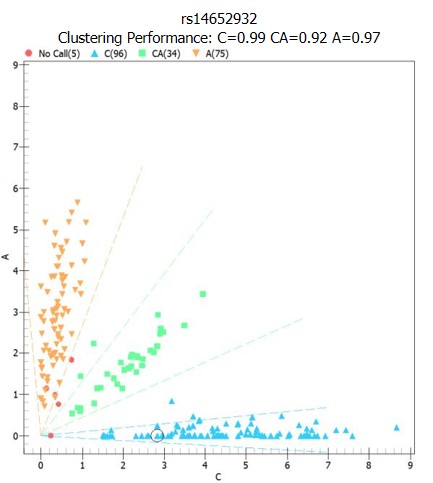

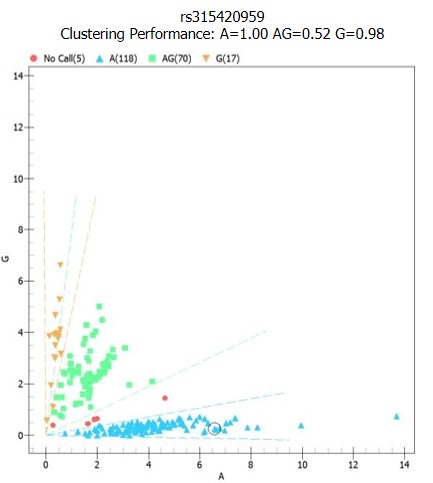

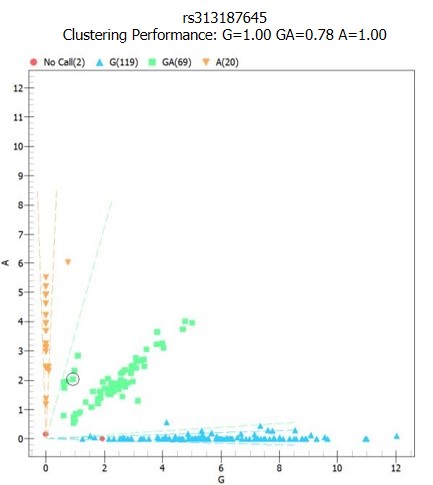

Supplement: Supplementary file 2 — Additional file 2: Figure S2. Mass spectrometry for 14 SNP markers associated with egg production traits. [file 12863_2020_815_MOESM2_ESM.docx]
