# Supplementary material for: Genotype frequency distributions of 28 SNP markers in two commercial lines and five Chinese native chicken populations
Source: BMC Genet. 2020 Feb 4;21:12. doi: 10.1186/s12863-020-0815-z (PMC7001339; doi:10.1186/s12863-020-0815-z)
Supplement: Supplementary file 3 — Additional file 3: Table S1. Go annotation of nearest genes associated with growth traits or carcass traits. [file 12863_2020_815_MOESM3_ESM.docx]

Additional file 3: Table S1. Go annotation of nearest genes associated with growth traits or carcass traits

| Gene name | Discription | Molecular function | Biological process |
| --- | --- | --- | --- |
| POU1F1 | POU class 1 homeobox 1; pituitary-specific transcription factor; growth hormone factor 1 | transcription factor that activates promoters of growth hormone (GH), prolactin (PRL) and thyroid-stimulating hormone-β (TSH-β) genes | positive regulation of transcription by RNA polymerase II |
| IGFBP2 | insulin-like growth factor-binding protein 2 | insulin-like growth factor I binding, insulin-like growth factor II binding | regulation of cell growth, regulation of insulin-like growth factor receptor signaling pathway |
| TBC1D1 | TBC1 domain family member 1 | Translocation of SLC2A4 (GLUT4) to the plasma membrane | unknown |
| ATGL | adipose triglyceride lipase; Patatin like phospholipase domain containing 2 | triglyceride lipase activity | lipid homeostasis, positive regulation of triglyceride catabolic process |
| OCA2 | oculocutaneous albinism II; OCA2 melanosomal transmembrane protein | unknown | cell proliferation, melanin biosynthetic process, melanocyte differentiation, spermatid development, transmembrane transport |
| CAPN3 | calpain-3; Calcium-activated neutral proteinase 3; calpain p94; muscle-specific calcium-activated neutral protease 3 | calcium-dependent cysteine-type endopeptidase activity, catalytic activity, structural constituent of muscle | cellular response to calcium ion, myofibril assembly, negative regulation of apoptotic process, positive regulation of NF-kappaB transcription factor activity, positive regulation of proteolysis, positive regulation of satellite cell activation involved in skeletal muscle regeneration, response to muscle activity |
| SLC27A1 | solute carrier family 27 member 1, fatty acid transport protein 1 | fatty acid transmembrane transporter activity, protein homodimerization activity | adiponectin-activated signaling pathway, cardiolipin biosynthetic process, long-chain fatty acid import, positive regulation of triglyceride biosynthetic process |
| PBEF1 | nicotinamide phosphoribosyltransferase, Visfatin, pre-B-cell colony-enhancing factor | nicotinate-nucleotide diphosphorylase (carboxylating) activity | NAD biosynthetic process |
| SETDB2 | SET domain bifurcated 2 | DNA binding, histone methyltransferase activity (H3-K9 specific), zinc ion binding | chromosome segregation, mitotic cell cycle |
| FOXO3 | forkhead box O3 | beta-catenin binding, chromatin DNA binding, RNA polymerase II transcription regulatory region sequence-specific DNA binding | cell differentiation, embryogenesis and morphogenesis, initiation of primordial ovarian follicle growth, antral ovarian follicle growth |
| BOD1L | biorientation of chromosomes in cell division 1 like 1 | unkown | cellular response to DNA damage stimulus, replication fork processing |
| MC4R | melanocortin 4 receptor | ubiquitin protein ligase binding, melanocyte-stimulating hormone receptor activity | feeding behavior, insulin secretion, regulation of metabolic process, positive regulation of bone resorption |
| FBXL5 | F-Box And Leucine Rich Repeat Protein 5 | ubiquitin protein transferase activity and iron ion binding. | protein catabolic process and iron ion homeostasis |
