# Supplementary material for: Genotype frequency distributions of 28 SNP markers in two commercial lines and five Chinese native chicken populations
Source: BMC Genet. 2020 Feb 4;21:12. doi: 10.1186/s12863-020-0815-z (PMC7001339; doi:10.1186/s12863-020-0815-z)
Supplement: Supplementary file 4 — Additional file 4: Table S2. Go annotation of nearest genes associated with reproduction traits. [file 12863_2020_815_MOESM4_ESM.docx]

Additional file 4: Table S2. Go annotation of nearest genes associated with reproduction traits

| Gene name | Discription | Molecular function | Biological process |
| --- | --- | --- | --- |
| HMGCR | 3-hydroxy-3-methylglutaryl coenzyme A reductase | hydroxymethylglutaryl-CoA reductase (NADPH) activity | cholesterol biosynthetic process, NADPH activity |
| BMP15 | Bone morphogenetic protein 15 | cytokine activity, growth factor activity | granulosa cell development, ovarian follicle development, regulation of BMP signaling pathway |
| OCX-32 | Ovocalyxin-32, retinoic acid receptor responder 1 | unknown | mineral deposition and eggshell completion |
| GREM1 | Gremlin-1 | bone morphogenetic proteins (BMPs) binding, cytokine activity, transmembrane receptor protein tyrosine kinase activator activity | cell morphogenesis, embryonic limb morphogenesis, regulation of BMP signaling pathway, branching involved in ureteric bud morphogenesis |
| GREM2 | Gremlin-2 | BMP binding, heparin binding | regulation of BMP signaling pathway, cytokine-mediated signaling pathway, embryonic body morphogenesis |
| KCNH7 | potassium voltage-gated channel subfamily H member 7 | voltage-gated potassium channel activity | regulation of ion transmembrane transport |
| CDC42BPA | CDC42 binding protein kinase alpha | ATP binding, metal ion binding, protein serine/threonine kinase activity | actin cytoskeleton organization, intracellular signal transduction |
| GJA5 | Gap junction alpha-5 | unknown | cell communication |
| STK31 | serine/threonine kinase 31 | ATP binding, nuclease activity, protein kinase activity | RNA catabolic process |
| CBFB | core-binding factor beta subunit | sequence-specific DNA binding, transcription coactivator activity | cell maturation, positive regulation of transcription by RNA polymerase II, myeloid cell differentiation, osteoblast differentiation, lymphocyte differentiation, definitive hemopoiesis |
| NCAPG | non-SMC condensin I complex subunit G | basic Helix-Loop-Helix (bHLH) transcription factor binding, methylated histone binding | erythrocyte differentiation, positive regulation of protein tyrosine kinase activity, regulation of transcription by RNA polymerase II |
| LCORL | ligand dependent nuclear receptor corepressor-like protein | DNA binding | regulation of transcription by RNA polymerase II, DNA-templated |
| FAF1 | fas associated factor 1 | NF-kappaB binding, protein domain specific binding, ubiquitin binding | cell death, positive regulation of extrinsic apoptotic signaling pathway via death domain receptors |
| SEL1L | ERAD E3 ligase adaptor subunit | unknown | protein secretion, triglyceride metabolic process, retrograde protein transport, ER to cytosol |
| GTF2A1 | General transcription facter IIA subunit 1 variant 1 | DNA binding, protein heterodimerization activity, RNA polymerase II repressing transcription factor binding | transcription by RNA polymerase II |
