# Supplementary material for: Genotype frequency distributions of 28 SNP markers in two commercial lines and five Chinese native chicken populations
Source: BMC Genet. 2020 Feb 4;21:12. doi: 10.1186/s12863-020-0815-z (PMC7001339; doi:10.1186/s12863-020-0815-z)
Supplement: Supplementary file 5 — Additional file 5: Table S3. The composition of PCR master mix. [file 12863_2020_815_MOESM5_ESM.docx]

Additional file 5: Table S3. The composition of PCR master mix

| PCR master mix of Reagent | Concentration | Volume |
| --- | --- | --- |
| Water, HPLC grade | NA | 1.850μl |
| PCR Buffer with 15mM MgCl2 | 1.25x | 0.625μl |
| MgCl2 (25mM) | 1.625mM | 0.325μl |
| dNTP Mix (25 mM each) | 500 μM | 0.100μl |
| Primer Mix (500 nM each) | 100uM | 1.000μl |
| HotStar Taq(5U/µl) | 0.5 U/rxn | 0.100μl |
| Total | - | 4.000μl |
