# Supplementary material for: Genotype frequency distributions of 28 SNP markers in two commercial lines and five Chinese native chicken populations
Source: BMC Genet. 2020 Feb 4;21:12. doi: 10.1186/s12863-020-0815-z (PMC7001339; doi:10.1186/s12863-020-0815-z)
Supplement: Supplementary file 6 — Additional file 6 Table S4. The composition of SAP mix. [file 12863_2020_815_MOESM6_ESM.docx]

Additional file 6: Table S4. The composition of SAP mix

| SAP mix of Reagent | Concentration | Volume |
| --- | --- | --- |
| Water (HPLC grade) | NA | 1.53μl |
| SAP Buffer | 10x | 0.17μl |
| SAP Enzyme | 1U/μl | 0.30μl |
| Total volume | - | 2.00μl |
