# Supplementary material for: Genotype frequency distributions of 28 SNP markers in two commercial lines and five Chinese native chicken populations
Source: BMC Genet. 2020 Feb 4;21:12. doi: 10.1186/s12863-020-0815-z (PMC7001339; doi:10.1186/s12863-020-0815-z)
Supplement: Supplementary file 7 — Additional file 7: Table S5. The composition of Extend mix. [file 12863_2020_815_MOESM7_ESM.docx]

Additional file 7: Table S5. The composition of Extend mix

| EXTEND Mix of Reagent | Concentration | Volume (1rxm) |
| --- | --- | --- |
| Water (HPLC grade) | NA | 0.619μl |
| iPLEX Buffer Plus | 0.222x | 0.200μl |
| iPLEX Termination mix | 1x | 0.200μl |
| Primer Mix (7 μM: 14 μM^1^) | 0.625 uM: 1.25uM | 0.200μl |
| iPLEX Enzyme | 1x | 0.041μl |
| Total volume | - | 2μl |

^1^ 7 μM: 14 μM: illustrate the doubled concentration of the high mass primers. Low mass, primers shoμld be at 0.625 uM and high mass primers at 1.25 μM in the final 9 μL reaction.
